# 

**Published:** 1899-06

**Authors:** 


### CONTENTS.

### COMMUNICATIONS.

"The Cuspid Tooth in its Relation to Character" ..... 251
Dental Ethics ..... 258
Bacteriology in the Treatment of Disease ..... 266

### PROCEEDINGS.

Southern Branch, National Dental Association, Second Annual Meeting, New Orleans, La., February 9, 10, 11 and 13, 1899 ..... 270
Microscopy, Histology and Bacteriology ..... 270
A Remarkable Case ..... 272
"Absorption Areas" and "Pulp Nodules" ..... 274
Formaldehyde in Dentistry ..... 275

### COMMENCEMENTS.

University of California—College of Dentistry ..... 279
Northwestern University Dental School ..... 280
Marion-Sims College of Medicine—Dental Department ..... 281
Chicago College of Dental Surgery ..... 281
Baltimore College of Dental Surgery ..... 283
Pennsylvania College of Dental Surgery ..... 283
Philadelphia Dental College ..... 284
University of Maryland—Department of Dental Surgery ..... 285
Cincinnati College of Dental Surgery ..... 286
Pittsburg Dental College ..... 287
University of Buffalo ..... 288
Southern Medical College—Dental Department ..... 288
Vanderbilt University, Department of Dentistry ..... 289
University of Omaha—Dental Department ..... 289
University of Tennessee, Dental Department ..... 290
University of Buffalo ..... 290
Kansas City Dental College ..... 291
Western Dental College ..... 291
Milwaukee Medical College—Dental Department ..... 292
Baltimore Medical College—Dental Department ..... 292
Central College of Dentistry ..... 292
Birmingham Dental College ..... 293
Illinois School of Dentistry ..... 293
University of Omaha ..... 293

### SELECTIONS.

A Bill to Provide for Registration in New South Wales ..... 294
Influence of Sunlight upon the Human Body ..... 295
Useful Hints ..... 296

### NOTICES.

Tennessee Dental Association ..... 296
The Sixteenth Annual Session of the National Association of Dental Examiners ..... 296
The Thirty-fifth Annual Meeting Missouri State Dental Association 297

### EDITORIAL.

Prosecution ..... 297
Michigan State Dental Society ..... 298
National Dental Association ..... 298
American Medical Association—Section on Stomatology ..... 299

### OBITUARY.

Dr. I. C. St. John ..... 300

